# A systematic review of influences on implementation of supported self-management interventions for people with severe mental health problems in secondary mental health care settings

**DOI:** 10.1371/journal.pone.0282157

**Published:** 2023-02-27

**Authors:** Samihah Islam, Rebecca Appleton, Chloe Hutchings-Hay, Brynmor Lloyd-Evans, Sonia Johnson

**Affiliations:** 1 Division of Psychiatry, University College London, London, United Kingdom; 2 Division of Psychiatry, NIHR Mental Health Policy Research Unit, University College London, London, United Kingdom; 3 Camden and Islington NHS Foundation Trust, London, United Kingdom; Universidad Internacional de La Rioja, SPAIN

## Abstract

**Purpose:**

There is robust evidence for offering supported self-management interventions for people with severe mental illness (SMI) throughout secondary mental health services, but their availability remains patchy. The aim of this systematic review is to synthesise the evidence on barriers and facilitators to implementing self-management interventions for people with SMI in secondary mental health care settings.

**Methods:**

The review protocol was registered with PROSPERO (CRD42021257078). Five databases were searched to identify relevant studies. We included full-text journal articles with primary qualitative or quantitative data on factors which affect the implementation of self-management interventions for people with SMI in secondary mental health services. The included studies were analysed using narrative synthesis, using the Consolidated Framework for Implementation Research and an established taxonomy of implementation outcomes.

**Results:**

Twenty-three studies from five countries met eligibility criteria. The barriers and facilitators identified in the review were mainly on the organisational level, but included some individual-level influences. Facilitators included high feasibility, high fidelity, a strong team structure, sufficient number of staff, support from colleagues, staff training, supervision, the presence of an implementation champion and adaptability of the intervention. Barriers to implementation include high staff turnover, staff shortage, lack of supervision, lack of support for staff delivering the programme, staff struggling with their increased workload, a lack of senior clinical leadership, and programme content perceived as irrelevant.

**Conclusion:**

The findings from this research suggest promising strategies to improve implementation of self-management interventions. For services providing support for people with SMI, organisational culture should be considered, as well as the adaptability of interventions.

## Introduction

Severe mental illness (SMI) has been defined in various ways but has often been used, as in this review, to refer to psychotic conditions, bipolar affective disorder and severe depressive disorder–conditions often associated with longer term use of mental health services [1]. Supported self-management interventions, hereafter referred to as self-management interventions, can be used to support the recovery of people with SMIs. These initiatives offer a chance to improve health care for this people with SMIs, as it can encourage adherence to treatment, help cope with symptom relapse and improve their broad quality of life [2].

Self-management can be defined in various ways, but generally involves the use of a range of techniques and tools and learning to actively manage one’s own health [3]. Approaches to achieving this include increasing knowledge of one’s condition (including medication management and administration), building new skills (including goal setting, coping with symptoms and making decisions about one’s own treatment plan) and increasing confidence in one’s ability to manage one’s condition (including creating a plan to recover more fully from one’s condition). Supported self-management involves a clinician, peer support worker or other supporter, who supports people in learning and applying such tools, with the aim that they may in future be able to do so independently [4]. Supported self-management interventions come in various forms, such as self-management education, peer support or health coaching, and can be delivered in various formats, such as group, individual or online [5].

Mueser and colleagues [6] identified the following effective components in self-management approaches: (a) psychoeducation about mental illness and its treatment (to make informed conditions about care); (b) recognition of early warning signs of relapse and development of a relapse prevention plan; (c) coping skills for dealing with persistent symptoms; and (d) strategies for medication management. Lean et al. [7] add to this list recovery-focused elements, such as setting personal goals and learning how to manage illness in order to pursue these goals. A widely used example of a self-management intervention is the Illness Management and Recovery package [8], which includes supporting people to find more social support and to discuss medication issues with clinicians, and development of individual relapse prevention plans and ways of coping with distressing symptoms.

Self-management support has been identified as a significant element in personalised care, empowering people to improve their self-confidence and quality of life, and to accomplish goals that are important to them [9]. Supported self-management has been shown to improve a range of outcomes among people with severe mental health problems [7]. A recent systematic review and meta-analysis [7] used some of the key elements, identified by Mueser and colleagues [6], to identify and investigate the effectiveness of self-management interventions for adults with SMI. Results show that the interventions demonstrated improvements for reducing symptoms and the length of hospital admission, and improving functioning and quality of life. Based on these findings, the researchers recommended that self-management interventions should be provided as standard components of mental health care for people with severe mental health problems. Reflecting this, health policies around the world emphasise the need to support people in managing their own health as effectively as possible [8,10]. For example, supported self-management is part of the NHS Long Term Plan’s commitment to integrate personalised care across the health and care system [5,11].

In the last two decades, supported self-management interventions have been the focus of increased interested (despite some doubts about whether they represent an unduly “medical” approach to mental health [12]), and have been introduced in many countries [13] and recommended in various clinical guidelines [14]. As well as their robust evidence base, the scope for a wide variety of professional and non-professional staff, including peer support workers, might facilitate widespread intervention. Despite this, availability and use of supported self-management interventions seem to be limited in many routine clinical settings [7]. This indicates a need for implementation research to understand how to promote integration of self-management interventions within mental health services.

Implementation refers to the process of incorporating and integrating evidence-based practices into real-life care settings [15]. The aim of implementation research is to achieve an understanding of whether and how these interventions work within everyday care settings, address challenges which arise as a result of incorporating these evidence-based interventions, and test ways to improve their uptake and delivery [16].

A recent narrative review [17] of self-management health interventions across multiple chronic conditions found that factors which can facilitate successful implementation included involving service users, engaging with local and business partners, involving stakeholders within the external system, tailoring the intervention, using multi-disciplinary teams, gathering feedback on effectiveness, having a feasible business model, adapting to organisational changes and anticipating changes that will be required within the healthcare system. An earlier synthesis [18] of factors influencing the adoption of self-management interventions for long-term illnesses identified factors related to patients, healthcare professionals and managers. Patient reports suggested that factors affecting whether they would adopt the intervention included their knowledge of their condition, their ability to adapt the strategies for daily use, visible effect of the intervention, and motivational factors. Healthcare professionals indicated that factors influencing their implementation of the intervention included whether it was evidence-based, integration of the intervention with the values of the service and with existing interventions, adaptability of the intervention, time and resource constraints, and motivational factors. For managers, factors included sustainable funding and resources, being able to deliver the intended benefits, good engagement with the business model, and senior leadership. It is unclear to what extent these findings are applicable to people with SMI in secondary mental health care settings–we are not aware of any previous systematic review that has focused on implementation of supported self-management in interventions for this group.

The aim of this review is therefore to synthesise the literature on the barriers and facilitators which influence the implementation of supported self-management interventions for people with SMI in secondary mental health care settings, such as community mental health services and outpatient clinics. As a secondary question, we also aimed to gather data on the outcomes of implementation.

## Methods

### Design

This systematic review follows Cochrane guidance [19] on conducting reviews and the Preferred Reporting Items for Systematic Reviews and Meta-Analyses (PRISMA) guidelines on reporting review processes [20]. The eligibility criteria for study inclusion were developed with the participants, interventions, comparators, outcomes, study design (known as PICOS) framework.

The review protocol was registered with PROSPERO (CRD42021257078).

### Search strategy

Five electronic databases (Embase, Medline, PsycInfo, CINAHL and Web of Science Core Collection) were utilised for the search. All databases were searched from their inception to the 29 May 2021 by the first author (SI). The search was limited to papers written in English. Additional searches were performed through hand-searching and forward citation searching of the included papers to ensure all relevant papers were identified.

An example search strategy for Ovid Embase is shown in S1 Appendix. Search terms were identified for four concepts: “self-management intervention”, “influencing factors”, “secondary care”, and “severe mental illness”. These terms were generated through conducting a limited search of the literature, analysing the keywords of the generated papers from this search, and by using the databases’ thesaurus to extend those keywords. Also, the terms were combined with standard MeSH terms from the PubMed and Cochrane databases and Subject Headings for the PsycINFO database.

All references were imported into EPPI-Reviewer 4 [21] for screening and selection, after de-duplication.

### Inclusion and exclusion criteria

**Inclusion criteria. Participants:** Staff facilitating the self-management intervention in a secondary mental health service, or people with a clinical diagnosis of non-affective psychosis (schizophrenia, schizophreniform disorder, schizoaffective disorder, delusional disorder, psychotic disorder not otherwise defined), bipolar disorder, or major depression who are receiving the intervention.

**Interventions:** The criteria used to define supported self-management were as in a previous review of effectiveness of supported self-management interventions conducted by members of the same research group [7]. To be included in the review, the intervention was required to include all of the following three domains, which are three of four domains that Mueser and colleagues [6] described as “effective areas of self-management”:

1. Psychoeducation about mental illness and its treatment
2. Recognition of early warning signs of relapse and development of a relapse prevention plan
3. Coping skills for dealing with persistent symptoms

**Comparator(s)/control:** A comparator or control group was not required.

**Exposures:** Any primary data on factors which influence the implementation of self-management interventions for people with SMI in secondary mental health services.

**Design:** Full-text journal articles with primary qualitative or quantitative data (or both). If the primary focus of the study was not an investigation of the barriers or facilitators to the use of self-management interventions but the results section included data relevant to the research question, the study was included.

#### Exclusion criteria

Articles were excluded if:

- The intervention did not focus on self-management of illness (e.g. the focus was on employment, physical health or social skills).
- The intervention focused on working with family members or carers of people with SMI.
- The intervention was delivered with or within another intervention (e.g. Individual Resilience Training within the NAVIGATE programme [22].
- The intervention was not conducted in a secondary mental health care setting (e.g. residential setting).
- The sample consisted of only participants with major depression, anxiety, personality disorders, or those with organic brain disorder or a primary diagnosis of substance abuse.
- They were commentaries, conference abstracts, review articles or studies without primary data.
- The article was published in a language other than English.

### Screening and selection

The titles and abstracts of the articles from the search were screened by the first author (SI) to identify studies which appear to meet the inclusion criteria. A second reviewer (CHH) screened 25% (1443) of titles and abstracts independently. The second reviewer (CHH) also screened 10% (561) of the studies excluded by the first author (SI). Agreement between reviewers at title and abstract screening was not calculated.

The full text of the included studies was independently reviewed for eligibility by the first author (SI) only. The reference lists of full-text articles selected were also screened by the first author (SI). After the included studies were determined, the second reviewer (CHH) screened all of the full texts of these studies. Any disagreements were resolved through discussion with other team members.

### Data extraction

A data extraction form was developed and piloted on five studies. Data extraction was conducted using EPPI-Reviewer 4 [21].

The data extraction form included details of study design, aims, gender, age, ethnicity, SMI diagnosis, study setting and context, intervention details, factors affecting implementation and implementation outcomes. To gather information related to implementation, a brief version of the Consolidated Framework for Implementation Research (CFIR) [23] was used, as well as an established taxonomy of implementation outcomes [24]. The CFIR gathered data related to factors potentially influencing implementation and the taxonomy of implementation outcomes gathered data related to factors ensuring successful implementation, in particular. These frameworks can help identify factors and aspects within implementation processes and create an evidence base around successful implementation. The two frameworks were combined to synthesise the factors which can influence implementation.

The CFIR [25] is a theoretical framework used to identify factors which influence implementation of interventions, within conceptual domains. The five major domains are: intervention characteristics (the perceived internal or external origin, evidence quality and strength, relative advantage compared to other interventions, trialability, adaptability, and complexity); outer setting (external influences such as organisational networks, peer pressure from other services and external policies and incentives); inner setting (characteristics of the implementing service including team culture, implementation climate, leadership engagement and networks and culture); characteristics of individuals, such as staff, involved in implementation (staff’s beliefs, knowledge, self-efficacy, and personal attributes which could affect implementation); and the implementation process (information on any stages of implementation, such as planning, executing, reflecting and evaluating, and any processes put in place to support implementation). The framework was adapted [23] to also include an additional domain on service user needs or resources; this domain covers the extent to which service user needs are known and feedback from service users about the intervention.

The taxonomy of implementation outcomes [24] is used to conceptualise and evaluate successful implementation. An intervention is unlikely to be effective if it is not implemented properly, so these outcomes indicate whether an intervention can be successfully put into practice and whether it is thus likely to achieve its goals in routine practice. The taxonomy includes the following concepts: acceptability, adoption, appropriateness, feasibility, fidelity, cost effectiveness, penetration and sustainability. Acceptability refers to satisfaction with the intervention. Adoption covers intention to try, uptake or utilisation of the intervention. Appropriateness refers to the perceived fit or compatibility of the intervention for the setting or service user. Feasibility is defined as the extent to which a new treatment can be successfully carried out within given setting. Fidelity is the degree to which an intervention was implemented as it was intended. Cost is defined as the cost impact of implementation. Penetration refers to the integration of an intervention within a service setting and its subsystems. Sustainability is the extent to which a newly implemented intervention is maintained within a service’s ongoing operations.

The first author (SI) independently extracted data from all included studies, and the second reviewer (CHH) independently extracted data from 20% of the included studies. Any disagreements were discussed with a third reviewer (RA).

### Data synthesis

The narrative synthesis began with an analysis of study characteristics, and further results were structured around the implementation of the interventions and the influencing factors. To guide the synthesis, the CFIR [23] and the taxonomy of implementation outcomes [24] was used. The frameworks provided a table with set categories (the defined domains) to extract relevant data into, and were used for data extraction by deductively coding findings from included studies to the domains. Data within each CFIR domain, and domains from the taxonomy of implementation outcomes, was inductively coded thematically; when conducting the synthesis with the frameworks, we identified any relevant themes across studies. We used a hybrid deductive-inductive approach for this analysis method.

### Study quality assessment

The quality of all included studies was assessed by the first author (SI) using the Mixed Methods Appraisal Tool (MMAT) [26], and the second reviewer (CHH) assessed 25% (6) of the included studies. The MMAT was used to inform us about the quality of information provided about implementation barriers and facilitators. The MMAT is a quality assessment tool which enables appraisal of qualitative, quantitative and mixed methods studies. The MMAT was scored by counting the fulfilled criteria. Qualitative and quantitative studies are appraised using five questions, and mixed methods studies using 15. The questions differ based on the study design and have been created to assess the methodological quality. Each study was assigned a percentage based on the score. Studies were not excluded based on the results of quality assessment.

## Results

### Study selection

**Fig 1:**
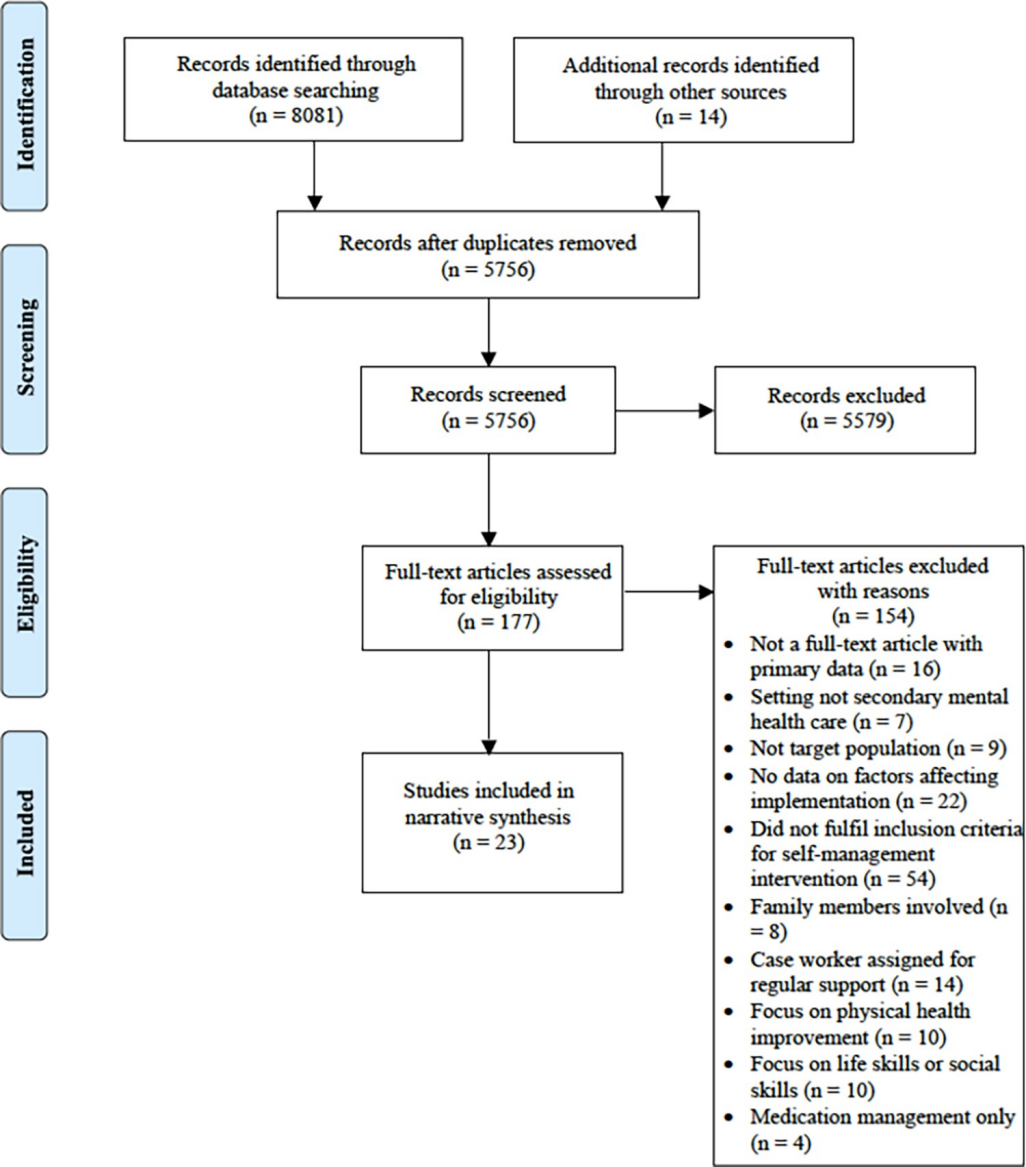
PRISMA diagram.

Searches of five electronic databases identified 8081 references, of which 23 studies met the inclusion criteria and were included in this review. No further articles were included by reviewing the reference lists of the included studies. Fig 1 shows the PRISMA diagram representing the stages of study selection.

### Study characteristics

Of the 23 included studies, three were categorised as qualitative, six as mixed methods, nine quantitative descriptive and five as quantitative randomised controlled trials (RCTs). The studies identified were published between 2006 and 2020.

All of the studies were conducted in community mental health services or outpatients in secondary mental health care settings. This distinction is made based on what the study authors described as the type of service. The authors did not explicitly state the difference between the two, or describe what the service entailed as this was often not relevant to the study being conducted. Four studies also had a service user led/peer support element [27–30]. The characteristics of the included studies are shown in Table 1.

**Table 1:** Study characteristics.

First author (year) Sample size (n)	Aim of the study	Study population	SMI diagnoses (% of sample)	Mean age (years) % female	Racial and ethnic composition of study sample	Country Type of service	Intervention details • Name • Duration • Brief description	Measurement methods used to collect outcome data
Bullock et al. (2006) [31] n = 35	Evaluation of the effectiveness of the IMR program in promoting mental health recovery	SUs	SZ (12.5%) BD (47.5%) MDD (40%)	42.2 years 60%	European American (84%) African American (10%) Latino or Hispanic American (3%) Other ethnicity (3%)	USA Community mental health service	• IMR programme • 5–6 months • A curriculum-based programme which uses psychoeducation, cognitive-behavioural methods for medication management, relapse prevention and coping skills training. The modules include recovery strategies, practical factors about schizophrenia, bipolar disorder and depression, treatment strategies, social support, medication management, relapse prevention, managing stress and persistent symptoms, getting your needs met and drug and alcohol use.	• Interview • Self-report measures
Carpenter-Song et al. (2020) [32] n = 15	Exploration of service user’s views on two interventions for people with serious mental illnesses	SUs	SZ (53%) BD (40%) MDD (7%)	NS 40%	White (33%) Black (60%) More than one race (7%)	USA Community mental health service	• WRAP • 12 weeks • A peer-led, group-based, curriculum-based approach. Sessions cover personal experiences, wellness tools, building management skills, daily maintenance, identifying relapse symptoms and managing crises.	• Interviews
Cook et al. (2009) [29] n = 80	Evaluation of the effectiveness of the WRAP program	SUs	SZ (20%) BD (38%) MDD (26%) PD (3%)	46.6 years 64%	Caucasian (66%) African American (25%) Hispanic or Latino (4%) Other ethnicity (5%)	USA Community mental health services; Outpatient services; Service user-led services	• WRAP • 8 weeks • Standard WRAP programme	• Interviews
Cook et al. (2012) [30] n = 251	Evaluation of the effectiveness of the WRAP program	SUs	SZ (21%) BD (38%) MDD (25%)	45.8 years 65.9%	Caucasian (63.2%) Black (28.1%) Hispanic/Latino (4.8%) Asian/Pacific Islander (0.6%) American Indian/Alaskan (2.9%) Other (0.4%)	USA Community mental health service; Service user-led services	• WRAP • 8 weeks • Standard WRAP programme	• Interview • Self-report measures
Coulthard et al. (2013) [33] n = 19 (SUs = 13)	Assessment of acceptability of a psychoeducation group for people with bipolar disorder	SUs Staff	BD (100%)	Group 1–44 years Group 2–36 years NS	NS	U.K. Community mental health service; Service user-led	• Bipolar psychoeducation group • 14 weeks • Structured group psychoeducation programme, involving homework, didactic teaching, interactive small group and individual exercises. Sessions covered early warning symptoms, medication, and lifestyle issues.	• Interviews • Group discussion • Observation • Self-report measures
de Andres et al. (2006) [34] n = 45	Exploration of impact of a structured group Life Goals Program on people with bipolar disorder	SUs	SZ (6.7%) BS (84.4%) Other: NS (8.9%)	NS 66.7%	Swiss (55.6%) Other European countries (33.3%)	Switzerland Outpatient services	• Life Goals Program • 6 weeks • Structured psychoeducational program which covers psychoeducation of bipolar disorders, depression and mania, identification of relapse symptoms, management strategies and an optional open-ended group related to goal setting with peers.	• Self-report measures
Enrique et al. (2020) [35] n = 18 (SUs = 15)	Assessment of acceptability and feasibility of an internet-delivered self-management intervention for people with bipolar disorder	SUs Staff	BD (100%)	40.23 years 23.1% (SUs)	NS	Ireland Community mental health service	• Bipolar Toolkit • 10 weeks • Internet-delivered programme for bipolar disorder. Modules cover personal recovery, goal setting, psychoeducation, treatment options, early warning signs, social support, and lifestyle issues.	• Interview • Self-report measures
Gottlieb et al. (2013) [36] n = 21	Assessment of acceptability and feasibility of a web-based cognitive-behavioural therapy for persons with psychosis	SUs	Schizophrenia (76%) Schizoaffective disorder (24%)	40.10 years 38%	Caucasian (57%) African American (38%) Asian (5%)	USA Community mental health service	• Coping with Voices • 10 weeks • Programme has ten lessons that are each designed to take 45 to 80 min to complete, depending on client speed. The lessons cover monitoring hallucinations, coping strategies and use of interactive games, and quizzes.	• Psychological test • Self-report measures
McGuire et al. (2013) [37] n = 60	Assessment of participation in IMR program	SUs	SZ (100%)	47.6 years 20.3%	White (33.9%)	USA Community mental health service; Outpatient service	• IMR programme • 9 months • Standard IMR programme	• Attendance • Interview • Self-report measures
McGuire et al. (2016) [38] n = 43	Evaluation of provider competence in providing IMR	Staff	SMI (NS)	NS 66.7%	NS	USA Community mental health service	• IMR programme • 3 months • Standard IMR programme	• Observation • Self-report measures
McHugo et al. (2007) [39] n = NS	Evaluation of fidelity in evidence-based practises	Staff	SMI (NS)	NS NS	NS	USA Community mental health service	• IMR programme • NS • Standard IMR programme	• Interviews • Observation
Monroe-DeVita et al. (2018) [40] n = 101	Assessment of feasibility of IMR within Assertive Community Treatment teams	SUs	SZ (91%) BD (19%)	43.9 years 41%	White (53%) Black (40%) Other ethnicity (7%)	USA Community mental health service	• IMR programme • 12 months • Standard IMR programme but with an additional 11th module on healthy living lifestyles	• Interviews • Self-report measures • Hospital admissions
Morse et al. (2020) [41] n = 72 (SUs = 17)	Evaluation of implementing IMR within Assertive Community Treatment teams	SUs Staff	SMI (NS)	NS 35%	African American (47%) White (47%) Saudi/African (6%)	USA Community mental health service	• IMR programme • 12 months • Standard IMR programme but with an additional 11th module on healthy living lifestyles	• Interview
Mueser et al. (2006) [8] n = 24	Evaluation of individual-based and group-based IMR	SUs	Schizophrenia (41.7%) Schizoaffective disorder (45.8%) BD (8.3%) Other: Delusional disorder (4.2%)	39.12 years 37%	Caucasian (89%) Black (11%)	USA + Australia Community mental health service; Outpatient service	• IMR programme • 9 months • Standard IMR programme delivered individually, and within groups	• Observation • Self-report measures
O’Connor et al. (2008) [42] n = 11	Exploration of service-users’ perspectives of a psychoeducation group	SUs	BD (100%)	41 years 64%	NS	UK Community mental health service	• Group psychoeducation • 8 weeks • Group psychoeducation. Sessions covered psychoeducation, relapse prevention, cognitive and behavioural strategies for managing depression and mania and coping with psychosocial stressors.	• Interview
Penn et al. (2011) [43] n = 46	Evaluation of acceptability and feasibility of the Graduated Recovery Intervention Program for first episode psychosis	SUs	P (100%)	22 years 39%	Caucasian (63%) African American (28%) Other (4, 9%)	USA Outpatient clinic	• Graduated Recovery Intervention Program • Up to 9 months • CBT programme for people who have experienced psychosis, involving engagement and wellness management, substance use, persistent symptoms and functional recovery. The sessions cover psychoeducation about psychosis, goal setting, illness management, dealing with persistent symptoms, and relapse prevention.	• Observation • Psychological test • Self-report measures
Richardson et al. (2019) [44] n = 23	Exploration of the impact of a CBT-based bipolar disorder psychoeducation group	SUs	BD (100%)	43 years 73.9%	White European (100%)	UK Community mental health service	• Cognitive behaviour therapy (CBT)-based bipolar disorder psychoeducation group • 12 weeks • A CBT-based BD psychoeducation group. Sessions covered mood monitoring, identifying symptoms, medication, CBT model, mindfulness, stress and anxiety management, lifestyle issues and interpersonal issues, and a Staying Well plan.	• Self-report measures and questionnaire
Salyers et al. (2009) [45] n = 324	Evaluation of the implementation of the IMR program	SUs	SMI (NS)	43.7 years 51%	Caucasian (82%)	USA Community mental health service	• IMR programme • NS • Standard IMR programme	• Observation • Self-report measures
Salyers et al. (2009) [27] n = 30 (SUs = 14)	Evaluation of a peer-led IMR program within a community mental health service	SUs Staff	SZ (73%) MDD (NS) BD (NS)	42.2 years 45% (SUs)	White (100%)	USA Community mental health service; Service user-led	• IMR programme • 9 months • Standard IMR programme delivered by a peer specialist	• Interview • Self-reported measures
Salyers et al. (2009) [28] n = 89	Evaluation of implementation of IMR using trainee perspectives	Staff	SMI (NS)	40.6 years 69.7%	NS	USA Community mental health service; outpatient services	• IMR programme • NS • Standard IMR programme	• Self-completed survey
Salyers et al. (2014) [46] n = 118	Evaluation of the effectiveness of IMR	SUs	SZ (100%)	47.76 years 20%	African American (61%) White (34%) More than one race (5%)	USA Community mental health service; outpatient services	• IMR programme • 9 months • Standard IMR programme	• Psychological test • Hospital admissions • Self-report measures
Tierney et al. (2011) [47] n = 157 (Cohort 1); 94 (Cohort 2)	Evaluation of treatment satisfaction of service users with serious mental illness participating in the Wellness Enhancement and Recovery Program	SUs	SZ (NS) MDD (NS) BD (NS) Other (NS)	NS NS (cohort 1); 54% (cohort 2)	Cohort 2: African American (62%) White (38%)	USA Community mental health service	• Wellness Enhancement and Recovery Program • NS • Uses the topics from IMR in a group setting, covers illness management and lifestyle issues	• Self-completed questionnaire
Whitley et al. (2009) [48] n = NS	Evaluation of implementing IMR in community mental health settings	Staff	SMI (NS)	NS NS	NS	USA Community mental health service	• IMR programme • 5–10 months • Standard IMR programme	• Interview • Observation

KEY: IMR, Illness Management and Recovery; WRAP, Wellness Recovery Action Planning; SU, service user; NS, not stated; SMI, severe mental illness; SZ, schizophrenia or schizoaffective disorder; BP, bipolar disorder; P, psychosis; MDD, major depressive disorder; PD, personality disorder.

Six (26%) of the 23 studies were primarily investigations of self-management interventions. The remainder had other primary goals, but reported data relevant to implementation: six (26%) were feasibility and acceptability trials, five (22%) were studies mainly assessing the effectiveness of interventions, four (17%) looked at perspectives of stakeholders and two (9%) were pilot studies.

The majority (18 out of 23) of included studies were conducted in USA, with one study [8] being conducted in both Australia and the USA. The other studies took place in the UK [32,42,44], Ireland [35] or Switzerland [34].

The majority of studies had samples including people with severe mental health problems of a mixture of types (including schizophrenia or schizoaffective disorder, depression and bipolar disorder) [27,28,30–32,38,39,45,48]. Three studies had a sample consisting of schizophrenia or schizoaffective disorder, depression and bipolar disorder, along with an “other” category [29,41,47]. The remaining studies had a sample made up of only people with psychosis [43], only people with schizophrenia or schizoaffective disorder [36,37,46], only people with bipolar disorder [33,35,42,44], people with schizophrenia or schizoaffective disorder and bipolar disorder [34,40] and one study had the former population makeup along with “other” [8].

The most common intervention within the included studies was the Illness Management and Recovery (IMR) programme [28,31,37–39,45,46,48]. The goals of the IMR programme are for people with SMI to learn about their mental illness and different treatment strategies, and to develop skills to alleviate symptoms, reduce relapses and progress towards recovery [8]. Strategies to achieve these goals include learning ways to increase social support, learning skills to discuss medication issues with clinicians, creating an individual relapse prevention plan, and identifying ways to cope with distressing symptoms. Two studies used the standard IMR programme with an additional module dedicated to healthy living lifestyles [40,41], one study delivered the programme individually and in groups [8] and one study employed a peer specialist to deliver the programme [27]. Three studies used the Wellness Recovery Action Planning (WRAP) programme [29,30,32]. The WRAP programme aims to help users develop skills to maintain wellbeing in their everyday life [49]. This involved creating a daily maintenance plan, understanding triggers, identifying early warning signs, creating an action plan, and crisis and post-crisis planning.

Other interventions include psychoeducation groups for people with bipolar disorder [33,34,42,44]; the Graduated Recovery Intervention Program (GRIP) for first episode psychosis [43], a cognitive behaviour therapy (CBT)-based programme for people who have experienced psychosis; the Wellness Enhancement and Recovery Program [47], which uses the topics from IMR in a group setting; an online self-management intervention for bipolar disorder [35] and the Coping with Voices programme for people with psychosis [36], an online CBT-based intervention.

Two of the studies described an implementation plan [45,48] and one study reported on the use of implementation model [39]. The aims of most studies were either evaluating the effectiveness or implementation of an intervention, or gathering service user or staff views of an intervention. Studies evaluating the effectiveness of an intervention were included as, although the focus of the study was not on barriers and facilitators to implementation, the outcomes measured did provide data relevant to our research questions, such as views of service users and evidence on implementation.

### Quality assessment

Of the 23 included studies, three were categorized as qualitative, 14 as quantitative and six as mixed methods. The quality of included primary research studies was mainly high, as 18 out of 23 studies met above 80% of quality criteria. Three studies were of moderate quality [8,33,44] and met between 60 and 75% of the criteria. Two low quality studies [37,40] met 40% of the criteria.

### Barriers and facilitators to self-management interventions

Findings on the barriers and facilitators to implementation were categorised with an adapted version of the CFIR framework [23]. The key findings have been summarised below.

#### Intervention characteristics

The most common modality used was face-to-face. These interventions included the IMR programme [8,27,28,31,37–41,45,46,48], WRAP [29,30,32], psychoeducation groups [33,42,44], Life Goals Program [34], GRIP [43], and WERP [47]. Two studies were web-based and used an online interface. These interventions were an internet-delivered self-management programme for bipolar disorder [35] and the Coping with Voices programme for people with psychosis [36]. The duration of the self-management interventions ranged from 6 weeks to 1 year. Interventions were supported by previously conducted studies displaying effectiveness, including RCTs [32,33,35,36,40,41,47]. In regards to the quality of the intervention and how it is presented, most participants had positive comments about the structure of the programme [27].

An important characteristic for improving implementation was reported to be adaptability of interventions and taking a flexible approach [41]. Being adaptable allows the programme to be tailored to service user and staff needs [33,36]. This included shortening the duration of the intervention sessions so participants felt more comfortable [41]. Other aspects included assessing whether further sessions of the intervention are required [43] and changing aspects of the programme to be more accessible and acceptable (e.g. language changes, larger font sizes, font colours) [8], which improved implementation as it allowed the programme to accommodate any challenges that arose. Having a flexible approach within the service was also reported to improve implementation [41]; adapting team meetings to discuss the intervention, and increasing communication between staff regarding the intervention ensured the process of implementation was efficient.

An advantage of web-based interventions compared to face-to-face support was found to be accessibility, since web-based interventions can be used in a variety of locations [35,36]. A relative advantage of peer-led group support was that it tended to be cost-effective [45], which was a facilitator to implementation. Services may find it easier to adopt interventions which have low costs.

#### Outer setting

External policies and incentives were often discussed in the introduction sections of articles explaining the rationale for interventions. A national enquiry in USA into the disparities between research and practice revealed gaps in the care for patients with schizophrenia. Therefore, the government developed reimbursement policies to support implementation of interventions in secondary mental health services [8,28]. Government organisations also sponsored the development of such interventions [35,45,46]. Government reports investigating the mental health care system have drawn attention to inadequate treatment and led to the recommendation of evidence-based and recovery focused treatment, including self-management interventions. Government policies in favour of self-management interventions were found to be a facilitator for implementation [45,47,48]. This was due to the funding and policy recommendations as it encouraged services to provide supported self-management interventions.

One study identified a concern that implementing a self-management intervention within an NHS cost-saving context would be difficult due to an inability to secure longer term funding for key staff posts [33]. Where staff members were required to support the intervention, as well as perform their regular duties; this was identified as a barrier to implementation.

#### Inner setting

Structural characteristics of organisations were a crucial factor for implementation. For example, a major challenge for implementation was high staff turnover, which in one study persisted despite support from leadership [45]. Another issue was a lack of infrastructure for supervision, coordination, and support for staff administering the intervention [45].

A facilitator for implementation was good staffing levels and a strong team structure–in this study, the intervention was delivered with the support of a nurse specialist, consultant psychiatrist and community mental health teams [33]. Each member had a designated role to ensure optimal implementation. An enabler, when there is a peer specialist involved, is a healthy collaboration between the health professional and the peer specialist, i.e. the health professional not always taking lead when the peer specialist displays low confidence [33]. Another facilitator was that the intervention was implemented in a stable, established service [27,32]. Provision of training, the materials of the intervention and support from colleagues or supervisors were the three most common facilitators reported by staff [28]. The availability of resources for training the staff and for recruitment of full-time staff to deliver the intervention improves implementation as the delivery of the intervention is aligned with the intended purpose [27].

Challenges to implementation include a lack of staff to provide practical support in delivering the intervention [35] and a particular pervasive issue is the health professional not being released from other duties they may have in their post, leading to an increased workload [33,35].

Leadership appeared to be important. A lack of support from senior clinical leaders can be a barrier since they can advocate for funding, especially when there are competing demands for resources [33]. One study found that strong leadership was present at all high-fidelity sites where the intervention was successfully implemented, however there was a lack of strong leadership at low-fidelity sites [48].

In the matter of networks and communications, some studies show that when the new intervention was not integrated well within the services, health professionals outside of the team implementing the service failed to respond appropriately to the service user’s needs [33,35]. For example, when the service user was experiencing a mental health crisis and showed the attending healthcare professional their action plan from the intervention, the plan was not acted upon by primary care, accident and emergency, and crisis resolution and home treatment teams.

Regarding organisational culture, it was reported that services with high-fidelity to the intervention displayed “a strong culture of innovation” and a generally positive attitude to new changes, whereas low-fidelity services were more “conserved” and displayed “organisational inertia” [48]. Staff at low-fidelity sites viewed new interventions as a burden, not an opportunity to improve the service.

#### Staff characteristics

Staff interviews from two studies revealed that staff generally had positive views about self-management interventions [27,35]. There was an issue of variability in formal knowledge about the diagnosis; it was found that peer specialists had less knowledge in some more technical areas [33]. However, as this was identified during training, the intervention providers could remedy this. Amongst peer specialists involved in the intervention, individual confidence varied–which was attributed by staff as due to lack of clinical training [27]. Peer specialists stated that, when facilitating sessions, it was difficult for them to meet the service users, who also had a diagnosis of bipolar disorder [33]. Some peer specialists dropped out, which affected the delivery of the intervention.

In one study, staff remained unenthusiastic and sceptical about the introduction of a new programme even after receiving training [8]. This is a barrier to implementation because if staff are not motivated to implement a new intervention, it is likely to affect the success of the intervention.

#### Process

A facilitator of implementation is training. The majority of studies described training schemes provided for staff; this included training courses from a specialist [8,27,33,35,38–41,46,48], detailed review of the intervention [30], phone consultations and conference calls [45], and didactic presentations and role plays [28,43]. Some training courses were for two days [8,28,30,40,48], or three days [33]. Provision of high-quality training by competent and respected trainers was found to be a key factor in some of the high-fidelity sites [48]. One evaluator noted training as an important facilitator at three of the top six sites and the other evaluator noted its importance at two of the top six sites. Training staff on the intervention is a facilitator because it will motivate staff to provide the intervention as intended, and provide them with clarity on the aims of the intervention, which will in turn increase their confidence.

Many studies involved staff, who were facilitating the self-management interventions, reflecting and evaluating the progress and quality of implementation [8,33,35,39,43,46]. This included supervision, weekly calls to ensure fidelity, and feedback on intervention sessions. One study used an organisational learning approach so after receiving feedback on implementation, the authors reflected and changed their approach [33]. This is a facilitator of implementation, as any challenges were addressed and dealt with immediately.

In some studies, it was reported to be helpful to identify and assign implementation champions within the service, who were responsible for managing implementation of the intervention [35,48]. These individuals were also responsible for inviting service users to join the intervention and keep up relationships [35]. Methods used to engage individuals involved in implementation included motivational kick-off meetings [45].

#### Service user needs

The success of the intervention is associated with the characteristics of the service delivering the intervention, and the content of the programme.

Both quantitative results from semi-structured questionnaires, using Likert scales, and interviews with service users show that positive experiences reported by the service users were related to helpful intervention content [27,32,33,35], and a strong sense of community within groups [32,33]. In addition, interviews found that positive experiences were related to intervention content being found relevant [31], a good relationship with the health professional [35,41,42] or peer specialist [27], and the presence of peer support [41].

Negative experiences reported by the service users in interviews, and potential barriers to successful implementation, included repetitive content [27,35], the intervention being too long for service users [31,41], and challenging group dynamics [32]. In interviews, some service users described difficulties in attending programmes in person [32] which reduced attendance [41], whereas others expressed difficulty with using online platforms [35].

Quantitative findings showed that higher educational level and less severe mental health problems were associated with higher attendance [37,41,46]. Other factors associated with higher attendance include older age and lower hostility [46]. In one study, 53% of staff involved in the implementation of the intervention reported barriers related to the service user, such as nonattendance, cognitive level, symptoms or motivation [28].

### Implementation outcomes

Findings on the outcomes of implementation were categorised using the Implementation Outcomes Taxonomy [24]. The key findings have been summarised below.

#### Acceptability

Acceptability refers to stakeholder’s satisfaction with the intervention [24]. Staff and service users generally had positive comments about self-management interventions, and viewed them as acceptable.

In interviews, staff delivering the interventions stated that they appreciated the value of a peer specialist [27], and the opportunity to improve their own clinical skills [41]. Questionnaire results show that staff appreciated a good facilitator training programme [33]–this refers to a training programme which involves all staff involved in the intervention, provides detailed instructions for delivering the intervention and has a flexible structure. In interviews, staff reported concerns about added workload and manging their role in the intervention, alongside their regular responsibilities [35,41,45].

Satisfaction surveys conducted revealed that the majority of service users found self-management programmes to be helpful [8,34,36,43] and were satisfied with their relationship with staff providing support with them [47].

Qualitative data was collected through conducting interviews with the service users. Service users found self-management programmes to be helpful [27,31,35], enjoyed the focus on the service users and individualisation [31], and appreciated being able to learn coping strategies [42]. The addition of a peer specialist [27] and use of a group format was also found to be acceptable, especially when there was more than one facilitator [31]. Not everyone appreciated a group experience however, as one study reported interpersonal issues between group members and some service users were not happy with other members’ level of commitment to the programme [32]. One study found that online self-management programmes were difficult to use for service users [35].

#### Adoption

There was little evidence regarding the adoption of self-management interventions in the included studies. One study reported no issues with uptake [40] and another described widespread adoption [8]. For one study, when staff were trained in the programme before implementation, it was found that not all staff trained subsequently participated [33]; in one case, less than half were involved in uptake [35].

#### Appropriateness

Service users in several studies reported that the intervention was a good fit for their needs [27,33,41] and the input from a peer specialist was valued by staff also [27]. Interventions were found to be fit for purpose, as they achieved the goals of teaching services users new skills and strategies [32]. Aspects which were rated at least ‘satisfactory’, and could be viewed as facilitators, include therapeutic relationship, recovery orientation, participation from all group members, and educational techniques [38].

There were concerns about the programme not being suitable for all individuals with SMI as the content could not be applied to everyone’s unique experiences [31] and as those who had been dealing with their diagnosis for several years were often already familiar with the content [35].

Staff implementing the intervention reported concerns about the intervention materials being too difficult to use, a lack of staff time and competing demands [28]. When staff competence was assessed, the average total from service user responses was found to be in the range of “needs improvement” [38]. The same study found that elements of programmes found to be unsatisfactory included weekly action plans which were not personalised, action plan follow-up, the cognitive-behavioural techniques (did not use modelling or role-playing a lot, as mainly reinforcement was used) and behavioural tailoring for medication management.

#### Feasibility

There is some evidence for feasibility, provided mainly by quantitative data. Feasibility can be measured through recruitment, retention and participation rates [50].

Studies stated that the intervention had good attendance and low drop-out rates [43] and that staff believe it was feasible [41]. Drop-out rates ranged from 11% to 32% [28,29,34,44–46]. Completion rates ranged from 46.6% to 77% [8,29,33,35]. Average attendance rate, measured by number of sessions attended, was 62% [30] and 78.54% [47].

#### Implementation cost

Studies did not evaluate the cost effectiveness of implementing the intervention, however two studies mentioned that one advantage of self-management intervention is the cost saving aspect, particularly when delivered in groups [33,34].

#### Penetration

Penetration, which looks at how well an intervention is integrated within a service, was not measured by many studies. Penetration level was determined to be low based on the extent to which the intervention is offered [45]. This was attributed to only one staff member providing the service. There were issues with integration of the intervention with other services provided in the organisation [41,46].

#### Sustainability

Two studies investigated the sustainability of the programme. One study reported the intervention had been expanded, with more staff members joining the team [27]. The other intervention was not implemented into routine practice in the longer term [33], although the reasons were not explicitly stated.

#### Fidelity

Fidelity was measured using weekly assessments via checklists [29,30], observations [30], and audiotaped sessions [43,46]. High fidelity was found by some studies [45]; in some cases, this was over 90% [29,30]. Moderately strong fidelity was also identified in three studies [27,43,46]. Fidelity was found to improve over time [45]. One study found that average scores for fidelity were below the threshold, and that interventions which require clinical skills had lower fidelity than those which did not [39]. When investigating fidelity, it was found that in high-fidelity sites, staff were committed to the recovery of service users, had previous experience with interventions requiring clinical skills and were generally more positive about implementing an innovative intervention [48]. On the other hand, at low-fidelity sites, there were staffing issues, such as high turnover, low pay, demoralisation and unsupportive leadership.

## Discussion

### Main findings

In this study, we conducted a systematic narrative review of the barriers and facilitators for implementing self-management interventions within secondary mental health settings. A total of 23 studies were included. To our knowledge, this review is the first of its kind to examine this topic.

The barriers and facilitators identified mainly related to organisational factors, with some being related to the individual. The narrative synthesis found some evidence to demonstrate feasibility of including self-management interventions in routine secondary mental health care, and high fidelity was a facilitator of implementation. Organisational facilitators of implementation included a strong team structure, sufficient number of staff available to support the intervention staffing, and support from colleagues. Factors directly related to the implementation process included the provision of training, supervision and the involvement of a designated implementation champion. Aspects of the intervention itself which could improve implementation include interventions being adaptable, the quality of the materials, and staff and service users perceiving the intervention content as relevant and helpful.

Barriers for implementation appeared to include organisational issues like a high staff turnover, staff shortage, lack of infrastructure for supervision and lack of support for staff delivering the intervention. A pervasive barrier was concerns over staff being expected to provide the intervention alongside their regular duties. Also, a lack of senior clinical leadership for the intervention was identified as a barrier to implementation. As mentioned previously, these barriers are related to organisational aspects, specifically readiness for implementation [25]. Readiness for implementation refers to indicators of organisational commitment to implementing an intervention and consists of leadership engagement, available resources and access to information. The identified barriers are generic ones that would potentially make it challenging to implement any new way of working.

### Comparisons to existing literature

The findings of our review, focused on mental health, are similar to systematic reviews examining implementation of self-management interventions in other settings, including physical health care settings. van Beest and colleagues [17] identified facilitating factors which included a tailored intervention that could adapt to organisational changes, and the involvement of multi-disciplinary teams. Whilst involvement of multi-disciplinary teams was not found to be a facilitator in our review, we did find that the lack of involvement from multiple disciplines was a barrier [33]. Additionally, they found that gathering feedback on effectiveness was a facilitator for implementation; similarly, in our review, one study [33] found that a facilitator for implementation was the use of an organisational learning approach whereby, after receiving feedback on implementation, the authors reflected and changed their approach. Furthermore, they reported that having a feasible business model facilitated implementation, and our review also found that having sufficient staffing and organisational resources was key to implementation. van Beest and colleagues [17] also reported that facilitators for implementation included the involvement of stakeholders such as service users, local and business partners, and anticipating required changes within the healthcare system. These were areas that seemed to have been little explored in the studies we included.

Common facilitators between our review and the synthesis conducted by Harvey and colleagues [18] include service user’s knowledge of their condition, adaptability of the intervention, and efficient integration of the intervention within the service. Common barriers identified include time and resource constraints, and a lack of support from senior leadership. In contrast, our review did not identify motivational factors as a facilitator, nor the visible effectiveness of the intervention. Furthermore, we did not identify sustainable funding and resources as a facilitator as there was a lack of data surrounding this topic.

### Implications

Our review makes the following recommendations after identifying several factors which could improve implementation. Firstly, developers of self-management interventions should consider adapting interventions to make them more inclusive for participants with less formal education lower education, as it was found that educational level was associated with attendance and therefore, a potential barrier in programmes which are curriculum-based. Other factors found to be associated with attendance included less mental health problem severity, and older age. This provides a basis to consider strategies for engaging service users who do not have the aforementioned attributes. In addition, the content of the intervention should be tailored to the service users and have flexibility to be personalised, since it was found that the intervention did not always fit their needs, and at times, service users were already very familiar with the content. To do this, developers could co-produce the content with potential service users; co-production can be used at each stage of the implementation process, including during the development of the implementation strategy. Alternatively, a novel self-management intervention could be created which is informed by the barriers and facilitators of implementation identified by this review. Also, interventions that require clinical skills from providers were difficult to implement for staff–this suggests that further training focused on clinical skills is required for staff facilitating the intervention.

The barriers and facilitators identified in this review have potential to form part of the basis for development of an implementation strategy for self-management interventions in secondary mental health care; this is likely to be most effective if co-produced and iteratively tested by a collaborative team including researchers, service users and clinicians. In the included studies, drop-out rates were found to range from 11% to 32%, and completion rates ranged from 46.6% to 77%; increasing these rates is a potential focus for implementation-focused interventions.

Based on the finding that several barriers are related to organisational readiness for change (ORC), service managers and stakeholders involved in implementing new self-management interventions could conduct an assessment of ORC [51] before introducing new interventions. This would allow identification of present barriers and provide implementers with the opportunity to address them. Services with less readiness for change may require more flexibility, or engagement with service leaders with the aim of addressing any remediable organisational barriers.

This review also identified current gaps in the literature which could provide focus for future research. For example, future research should explore the cost-effectiveness of implementing self-management interventions, as well as penetration and sustainability, as the studies included in this review did not contain much information on these areas of implementation.

### Strengths and limitations

To our knowledge, this is the first systematic review mental health self-management interventions for SMI in secondary care. A strength of this review is the use of the CFIR and established taxonomy to analyse the results. This allowed us to translate the quantitative and qualitative evidence in a broader context for policy development and evidence-informed practice. Furthermore, the majority of the included studies were of high quality.

While the findings have implications for informing care delivery for people with SMI, there are some limitations that must be considered. Firstly, only articles published in the English language were included, therefore, some relevant literature published in other languages may have been omitted. Secondly, although exhaustive search methods were used, some studies may have been missed, especially since grey literature was not searched. However, the likelihood of publication status being a significant source of bias is low, especially in the context of this topic. We were looking at full-text journal articles with primary data, because to accurately gauge influence on implementation of self-management interventions for people with SMI, we believe a rigorous study would need to be conducted. Thirdly, all of the included studies were conducted in high-income countries, which limits the generalisability of the findings. Mental health services may be less developed in low- and middle-income countries [52], due to scarce resources for mental health. Thus implementation barriers may be greater in these countries, although supported self-management may be potentially valuable given the relatively low levels of resource and staff training that tend to be involved [53]. Due to differences in health care service delivery, future research is required to explore the factors which affect implementation of self-management interventions in low- and middle-income countries. Another limitation to consider is that only a minority of the included studies actually focused on implementation. Only three studies were explicitly based on implementation science theories and models [39,45,48]. Whilst the other studies did provide relevant data related to implementation, the richness and completeness of data is likely to be less than for research based on implementation science. A further limitation of this study is that whilst we collected data on the study setting, we did not investigate whether there is variation in secondary care settings as this is beyond the scope of the project. Typically, community mental health services will be multi-disciplinary and based in non-hospital settings [54], whereas outpatient clinics may only involve care from a psychiatrist and are typically hospital-based [55]; however, there may be considerable variation and some overlap within these categories. This should be addressed in future research. Finally, more than one reviewer did not double-screen, or extract data from all of the studies. Only one reviewer coded all of the identified barriers and facilitators, which could potentially cause bias as qualitative data is subjective [56]. An attempt to remedy this was taken by having a second reviewer also review a portion of the included studies at each step.

## Conclusion

The area of self-management interventions for SMI has potential to improve health care services. The findings of this systematic review suggest some potentially promising strategies to improve the implementation of these interventions, but they also highlight important gaps that future research should address. Overall, more facilitators than barriers were reported. Many factors were related to organisational determinants and some on individual level determinants, which highlights a need for a better understanding of contextual determinants. Identifying these factors provides a starting point for developing implementation strategies for self-management interventions for people with SMI.

## Supporting information

S1 Checklist Preferred Reporting Items for Systematic Reviews and Meta-Analyses (PRISMA) checklist. (DOC)

S1 Appendix Example search strategy using Embase. (DOCX)

S1 File Registered protocol. This is the review protocol, which was registered with PROSPERO.
(PDF)

## References

[1] Guidelines for the management of physical health conditions in adults with severe mental disorders. Geneva: World Health Organization; 2018. Available from: https://www.ncbi.nlm.nih.gov/books/NBK534487/. 30507109

[2] Evans TS, Berkman N, Brown C, Gaynes B, Weber RP. Disparities within serious mental illness. Rockville: Agency for Healthcare Research and Quality (US) [Internet]; 2016 May [cited 2021 July 20]. Available from: https://www.ncbi.nlm.nih.gov/books/NBK368427/. 27336120

[3] Recovery Davidson L., self management and the expert patient–changing the culture of mental health from a UK perspective. J Ment Health. 2005;14(1): 25–35.

[4] Taylor S, Pinnock H, Epiphaniou E, Pearce G, Parke H, Schwappach A et al. A rapid synthesis of the evidence on interventions supporting self-management for people with long-term conditions (PRISMS Practical systematic RevIew of Self-Management Support for long-term conditions). Health Serv Deliv Res. 2014;2(53): 1–580. 25642548

[5] National Health Service (NHS). Supported self-management: summary guide [Internet]. England: NHS. 2020 [cited 2021 July 20] Available from: https://www.england.nhs.uk/publication/supported-self-management-summary-guide/.

[6] Mueser KT, Corrigan P, Hilton D, Tanzman B, Schaub A, Gingerich S et al. Illness management and recovery: a review of the research. Psychiatr. Serv. 2002;53(10): 1272–1284. doi: 10.1176/appi.ps.53.10.1272 12364675

[7] Lean M, Fornells-Ambrojo M, Milton A, Lloyd-Evans B, Harrison-Stewart B, Yesufu-Udechuku A et al. Self-management interventions for people with severe mental illness: systematic review and meta-analysis. Br J *Psychiatry*. 2019;214(5): 260–268. doi: 10.1192/bjp.2019.54 30898177 PMC6499726

[8] Mueser KT, Meyer PS, Penn DL, Clancy R, Clancy DM, Salyers MP. The illness management and recovery program: rationale, development, and preliminary findings. Schizophr. Bull. 2006;32(Supplement 1): S32–S43. doi: 10.1093/schbul/sbl022 16899534 PMC2685197

[9] Voices National. Prioritising person-centred care: supporting self-management–summarising evidence from systematic reviews [Internet]. England: National Voices. 2014 [cited 2021 July 20] Available from: https://www.nationalvoices.org.uk/publications/our-publications/supporting-self-management.

[10] Anya DL PF, Julie F, Lisa K. A practical guide to self-management support: key components for successful implementation [Internet]. England: The Health Foundation. 2015 [cited 2021 July 20]. Available from: https://www.health.org.uk/publications/a-practical-guide-to-self-management-support.

[11] National Health Service (NHS). The NHS long term plan [Internet]. England: NHS. 2019 [cited 2021 July 20]. Available from: https://www.longtermplan.nhs.uk/.

[12] Bilsker D. Self-management in the mental health field. Visions [Internet]. 2003 [cited 2021 July 20];1(18): 4–5. Available from: https://www.heretohelp.bc.ca/self-management-mental-health-field.

[13] eMEN North West Europe. e-mental health innovation and transnational implementation platform North West Europe (eMEN). 2022 May 18 [Cited 03 Feb 2023]. Available: http://www.nweurope.eu/projects/project-search/e-mental-health-innovation-and-transnational-implementation-platform-north-west-europe-emen/.

[14] National Institute for Health and Care Excellence (NICE). Psychosis and schizophrenia in adults: prevention and management [Internet]. England: NICE. 2014 [cited 2021 July 20]. Available from: https://www.nice.org.uk/guidance/cg178. 32207892

[15] May C, Johnson M, Finch T. Implementation, context and complexity. Implement Sci. 2016;11(1): 141. doi: 10.1186/s13012-016-0506-3 27756414 PMC5069794

[16] Peters D, Adam T, Alonge O, Agyepong I, Tran N. Republished research: implementation research: what it is and how to do it. Br J Sports Med. 2014;48(8): 731–736. 24659611 10.1136/bmj.f6753

[17] van Beest W, Boon W, Andriessen D, Moors E, van der Veen G, Pol H. Successful implementation of self-management health innovations. J Public Health (Berl.). 2020;30(3):721–735.

[18] Harvey J, Dopson S, McManus R, Powell J. Factors influencing the adoption of self-management solutions: an interpretive synthesis of the literature on stakeholder experiences. Implement Sci. 2015;10(1): 1–15. doi: 10.1186/s13012-015-0350-x 26566623 PMC4644277

[19] Higgins JPT TJ, Chandler J, Cumpston M, Li T, Page MJ, Welch VA, editors. (2021) Cochrane handbook for systematic reviews of interventions version 6.2 (updated February 2021) [Internet]. England: Cochrane. 2021 [cited 2021 July 20]. Available from www.training.cochrane.org/handbook.

[20] Moher D, Liberati A, Tetzlaff J, Altman D. Preferred reporting items for systematic reviews and meta-analyses: the PRISMA statement. BMJ. 2009;339: b2535–b2535. doi: 10.1136/bmj.b2535 19622551 PMC2714657

[21] Thomas J, Graziosi S, Brunton J, Ghouze Z, O’Driscoll P, Bond M. EPPI-Reviewer: advanced software for systematic reviews, maps and evidence synthesis. Computer software. EPPI-Centre Software, UCL Social Research Institute London. Available from: https://eppi.ioe.ac.uk/CMS/Default.aspx?alias=eppi.ioe.ac.uk/cms/er4&

[22] Mueser K, Meyer-Kalos P, Glynn S, Lynde D, Robinson D, Gingerich S et al. Implementation and fidelity assessment of the NAVIGATE treatment program for first episode psychosis in a multi-site study. Schizophr Res. 2019;204: 271–281. doi: 10.1016/j.schres.2018.08.015 30139553 PMC6382606

[23] Safaeinili N, Brown‐Johnson C, Shaw J, Mahoney M, Winget M. CFIR simplified: pragmatic application of and adaptations to the Consolidated Framework for Implementation Research (CFIR) for evaluation of a patient‐centered care transformation within a learning health system. Learn Health Syst. 2019;4(1): e10201. doi: 10.1002/lrh2.10201 31989028 PMC6971122

[24] Proctor E, Silmere H, Raghavan R, Hovmand P, Aarons G, Bunger A et al. Outcomes for implementation research: conceptual distinctions, measurement challenges, and research agenda. Adm Policy Ment Health. 2010;38(2): 65–76. 10.1007/s10488-010-0319-7 PMC3068522 20957426

[25] Damschroder L, Aron D, Keith R, Kirsh S, Alexander J, Lowery J. Fostering implementation of health services research findings into practice: a consolidated framework for advancing implementation science. Implement Sci. 2009;4(1). 10.1186/1748-5908-4-50 PMC2736161 19664226

[26] Hong QN, Fàbregues S, Bartlett G, Boardman F, Cargo M, Dagenais P et al. The Mixed Methods Appraisal Tool (MMAT) version 2018 for information professionals and researchers. Educ. Inf. 2018;34(4): 285–291.

[27] Salyers M, Hicks L, Mcguire A, Baumgardner H, Ring K, Kim H. A pilot to enhance the recovery orientation of assertive community treatment through peer-provided illness management and recovery. Am. J. Psychiatr. Rehabil. 2009;12(3): 191–204.

[28] Salyers MP, Rollins AL, McGuire AB, Gearhart T. Barriers and facilitators in implementing illness management and recovery for consumers with severe mental illness: trainee perspectives. Adm Policy Ment Health. 2009;36 (2): 102–111. doi: 10.1007/s10488-008-0200-0 19096924

[29] Cook J, Copeland M, Hamilton M, Jonikas J, Razzano L, Floyd C et al. Initial outcomes of a mental illness self-management program based on wellness recovery action planning. Psychiatr Serv. 2009;60(2): 246–249. doi: 10.1176/ps.2009.60.2.246 19176420

[30] Cook JA, Copeland ME, Jonikas JA, Hamilton MM, Razzano LA, Grey DD et al. Results of a randomized controlled trial of mental illness self-management using wellness recovery action planning. Schizophr Bull. 2012;38 (4): 881–891. doi: 10.1093/schbul/sbr012 21402724 PMC3406522

[31] Bullock WA ORM, Breedlove A, Farrer E, Smith MK. Effectiveness of the illness management and recovery program in promoting recovery: preliminary results. New Research in Mental Health. 2006;17: 282–291

[32] Carpenter-Song E, Jonathan G, Brian R, Ben-Zeev D. Perspectives on mobile health versus clinic-based group interventions for people with serious mental illnesses: a qualitative study. Psychiatr Serv. 2020;71(1): 49–56. doi: 10.1176/appi.ps.201900110 31615368

[33] Coulthard K, Patel D, Brizzolara C, Morriss R, Watson S. A feasibility study of expert patient and community mental health team led bipolar psychoeducation groups: implementing an evidence based practice. BMC Psychiatry. 2013;13: 301–301. doi: 10.1186/1471-244X-13-301 24215655 PMC3830443

[34] de Andrés RD, Aillon N, Bardiot MC, Bourgeois P, Mertel S, Nerfin F et al. Impact of the life goals group therapy program for bipolar patients: an open study. J Affect Disord. 2006;93(1–3): 253–257. doi: 10.1016/j.jad.2006.03.014 16675029

[35] Enrique A, Duffy D, Lawler K, Richards D, Jones S. An internet-delivered self-management programme for bipolar disorder in mental health services in Ireland: results and learnings from a feasibility trial. Clinical Psychol Psychother. 2020;27(6): 925–939. 10.1002/cpp.2480 PMC7754375 32445611

[36] Gottlieb JD, Romeo KH, Penn DL, Mueser KT, Chiko BP. Web-based cognitive-behavioral therapy for auditory hallucinations in persons with psychosis: a pilot study. Schizophr Res. 2013;145(1–3): 82–87. doi: 10.1016/j.schres.2013.01.002 23410709

[37] McGuire AB, Bonfils KA, Kukla M, Myers L, Salyers MP. Measuring participation in an evidence-based practice: illness management and recovery group attendance. Psychiatry Res. 2013;210(3): 684–689. doi: 10.1016/j.psychres.2013.08.008 24011850 PMC3971633

[38] McGuire AB, Bartholomew T, Anderson AI, Bauer SM, McGrew JH, White DA et al. Illness management and recovery in community practice. Psychiatr Rehabil J. 2016;39(4): 343–351. doi: 10.1037/prj0000200 27505349 PMC5125841

[39] McHugo GJ, Drake RE, Whitley R, Bond GR, Campbell K, Rapp CA et al. Fidelity outcomes in the National Implementing Evidence-Based Practices Project. Psychiatr Serv. 2007;58(10): 1279–1284. doi: 10.1176/ps.2007.58.10.1279 17914003

[40] Monroe-DeVita M, Morse G, Mueser KT, McHugo GJ, Xie H, Hallgren KA et al. Implementing Illness Management and Recovery within assertive community treatment: a pilot trial of feasibility and effectiveness. Psychiatr Serv. 2018;69(5): 562–571. doi: 10.1176/appi.ps.201700124 29446335 PMC6433370

[41] Morse G, Monroe-DeVita M, York MM, Peterson R, Miller J, Hughes M et al. Implementing illness management and recovery within assertive community treatment teams: a qualitative study. Psychiatr Rehabil J. 2020;43(2): 121–131. doi: 10.1037/prj0000387 31478709 PMC7050388

[42] O’Connor C, Gordon O, Graham M, Kelly F, O’Grady-Walshe A. Service user perspectives of a psychoeducation group for individuals with a diagnosis of bipolar disorder: a qualitative study. J Nerv Ment Dis. 2008;196 (7): 568–571. doi: 10.1097/NMD.0b013e31817d0193 18626298

[43] Penn DL, Uzenoff SR, Perkins D, Mueser KT, Hamer R, Waldheter E et al. A pilot investigation of the Graduated Recovery Intervention Program (GRIP) for first episode psychosis. Schizophr Res. 2011;125(2–3): 247–256. doi: 10.1016/j.schres.2010.08.006 20817484 PMC3010489

[44] Richardson T, White L. The impact of a CBT-based bipolar disorder psychoeducation group on views about diagnosis, perceived recovery, self-esteem and stigma. The Cognitive Behaviour Therapist. 2019;12: e43.

[45] Salyers MP, Godfrey JL, McGuire AB, Gearhart T, Rollins AL, Boyle C. Implementing the illness management and recovery program for consumers with severe mental illness. Psychiatr Serv. 2009;60(4): 483–490. doi: 10.1176/ps.2009.60.4.483 19339323

[46] Salyers MP, McGuire AB, Kukla M, Fukui S, Lysaker PH, Mueser KT. A randomized controlled trial of illness management and recovery with an active control group. Psychiatr Serv. 2014;65(8): 1005–1011. doi: 10.1176/appi.ps.201300354 24733680

[47] Tierney KR, Kane CF Promoting wellness and recovery for persons with serious mental illness: a program evaluation. Arch Psychiatr Nurs. 2011;25(2): 77–89. 21421159 10.1016/j.apnu.2010.07.006

[48] Whitley R, Gingerich S, Lutz WJ, Mueser KT. Implementing the illness management and recovery program in community mental health settings: facilitators and barriers. Psychiatr Serv. 2009;60(2): 202–209. doi: 10.1176/ps.2009.60.2.202 19176414

[49] Copeland ME. Wellness Recovery Action Plan. Occup Ther Ment Health. 2002;17(3–4): 127–150.

[50] McGuire AB, Kukla M, Green A, Gilbride D, Mueser KT, Salyers MP. Illness management and recovery: a review of the literature. Psychiatr Serv. 2014;65(2): 171–179. doi: 10.1176/appi.ps.201200274 24178191 PMC4203303

[51] Hamilton AB, Cohen AN, Young AS. Organizational readiness in specialty mental health care. J Gen Intern Med. 2010;25(1): 27–31. doi: 10.1007/s11606-009-1133-3 20077148 PMC2806963

[52] Rathod S, Pinninti N, Irfan M, Gorczynski P, Rathod P, Gega L et al. Mental health service provision in low- and middle-income countries. Health Serv Insights. 2017;10: 1178632917694350. doi: 10.1177/1178632917694350 28469456 PMC5398308

[53] Eaton J, McCay L, Semrau M, Chatterjee S, Baingana F, Araya R et al. Scale up of services for mental health in low-income and middle-income countries. Lancet. 2011;378(9802): 1592–1603. doi: 10.1016/S0140-6736(11)60891-X 22008429

[54] NHS. The community mental health framework for adults and older adults. 2019. Available from: https://www.england.nhs.uk/wp-content/uploads/2019/09/community-mental-health-framework-for-adults-and-older-adults.pdf.

[55] NHS. Choice in mental health care. 2021. Available from: https://www.england.nhs.uk/wp-content/uploads/2018/02/choice-in-mental-health-care-v5.pdf.

[56] Waffenschmidt S, Knelangen M, Sieben W, Bühn S, Pieper D. Single screening versus conventional double screening for study selection in systematic reviews: a methodological systematic review. BMC Med Res Methodol. 2019;19(1): 132. doi: 10.1186/s12874-019-0782-0 31253092 PMC6599339

