# Supplementary material for: A systematic review of influences on implementation of supported self-management interventions for people with severe mental health problems in secondary mental health care settings
Source: PLoS One. 2023 Feb 27;18(2):e0282157. doi: 10.1371/journal.pone.0282157 (PMC9970054; doi:10.1371/journal.pone.0282157)
Supplement: S1 Appendix — (DOCX) [file pone.0282157.s002.docx]

Supporting information

S2 Table. Example search string using Embase

Platform Ovid Database

Medline limits DATE; Human; English

| Database: Embase <1974 to 2021 May 29> |
| --- |
| 1 exp schizophrenia/ (184195)  2 exp bipolar disorder/ or bipolar disorder*.ti,ab,kw. (74320)  3 exp psychosis/ (288061)  4 exp Depression/ or major depressive disorder.ti,ab,kw. (506863)  5 ((chronic* or serious* or sever*) adj2 mental* adj2 (ill* or disorder*)).ti,ab,kw. (17715)  6 (delusional disorder* or paranoid disorder* or hebephrenic* or oligophreni* or psychos*s or psychotic* or schizo*).ti,ab,kw. (258385)  7 akathisia/ or dyskinesia/ or neuroleptic malignant syndrome/ (30874)  8 (akathisi* or acathisi* or (neuroleptic* and ((malignant and syndrome) or (movement adj2 disorder))) or (tardiv* and dyskine*)).ti,ab,kw. (11793)  9 (parkinsoni* or neuroleptic induc*).ti,ab,kw. not (parkinson* and disease).ti. (30986)  10 1 or 2 or 3 or 4 or 5 or 6 or 7 or 8 or 9 (839355)  11 exp self care/ or exp self evaluation/ or exp self help/ (120101)  12 ((self adj (administer* or assess* or attribute* or care or change or directed or evaluat* or efficacy or help* or guide* or instruct* or manag* or medicat* or monitor* or regulat* or reinforc* or re inforc* or support* or technique* or therap* or train* or treat*)) or selfadminister* or selfassess* or selfattribut* or selfcare or selfchange or selfdirected or selfefficacy or selfhelp* or selfguide* or selfinstruct* or selfmanag* or selfmedicat* or selfmonitor* or selfregulat* or selfreinforc* or self re inforc* or selfsupport* or selftechnique* or selftherap* or selftrain* or selftreat*).ti,ab. (222141)  13 (expert patient* or (hearing voices adj2 (group* or network* or support*)) or (minimal adj (contact or guidance)) or helpseek* or (help* adj2 seek*) or (mutual adj (aid* or help or support*)) or recovery model* or smart recovery).ti,ab. (18518)  14 (booklet* or brochure* or leaflet* or pamphlet* or poster* or psychoeducat* or psycho educat* or workbook* or work book* or ((adult* or client* or consumer* or health or inpatient* or outpatient* or participant* or patient* or service user*) adj2 (educat* or focus* or information* or knowledge or learn* or literac* or promot* or taught or teach*)) or empower* or ((oral or printed or written) adj3 (material* or inform*))).ti,ab. (890760)  15 exp consumer health information/ or health education/ or health literacy/ or health promotion/ or patient education/ or psychoeducation/ (321022)  16 adaptive behavior/ (52201)  17 (((behav* or psychologic*) adj3 (adapt* or adjust*)) or cope or copes or coping).ti,ab. (138790)  18 patient participation/ (29335)  19 ((adult* or client* or consumer* or inpatient* or outpatient* or participant* or patient* or service user*) adj2 (involv* or participat*)).ti,ab. (129239)  20 Recovery.ti,ab,kw. (627883)  21 11 or 12 or 13 or 14 or 15 or 16 or 17 or 18 or 19 or 20 (2188341)  22 (Implement* or attitude*).ti,ab,kw. (905274)  23 health personnel attitude/ or attitude to health/ or consumer attitude/ or patient attitude/ or employee attitude/ (256794)  24 (User* participation* or patient* participation* or patient* perspective* or user* perspective* or patient* percep* or health perception* or user* percep* or user* view* or patient* view* or patient* prefer*).ti,ab,kw. (72416)  25 (choice* or value* or valuation*).ti,ab,kw. (3101789)  26 (acceptab* or expectation* or knowledge or point of view or preference*).ti,ab,kw. (1550121)  27 (Barrier* or Belief* or Cause* or decision* or deliver* or determinant* or enable* or Encourag* or Facilitat* or Facilitating factor* or Hurdle* or implement* or Obstruct* or opinion* or Perceived challenge* or perception* or promot* or support*).ti,ab,kw. (9525739)  28 Program evaluation/ or program evaluation.ti,ab,kw. (20889)  29 22 or 23 or 24 or 25 or 26 or 27 or 28 (12529885)  30 secondary health care/ or secondary care.ti,ab,kw. (14664)  31 Secondary Prevention/ or secondary prevention.ti,ab,kw. (44436)  32 community care/ (55965)  33 (secondary health* or community mental health* or crisis resolution team or assertive community treatment team or community care* or community-based mental health service*).ti,ab,kw. (16457)  34 30 or 31 or 32 or 33 (126194)  35 10 and 21 and 29 and 34 (1715)  36 limit 35 to english language (1633) |
