# Supplementary material for: A systematic review of influences on implementation of supported self-management interventions for people with severe mental health problems in secondary mental health care settings
Source: PLoS One. 2023 Feb 27;18(2):e0282157. doi: 10.1371/journal.pone.0282157 (PMC9970054; doi:10.1371/journal.pone.0282157)
Supplement: S1 File — This is the review protocol, which was registered with PROSPERO. (PDF) [file pone.0282157.s003.pdf]

Citation

Samihah Islam, Rebecca Appleton, Sonia Johnson, Bryn Lloyd-Evans, Chloe Hutchings-Hay, Wing Tung Chung. Factors impacting the implementation of self-management interventions in secondary care for people with severe mental illness (SMI): a systematic review. PROSPERO 2021 CRD42021257078 Available from: https://www.crd.york.ac.uk/prospero/display_record.php?ID=CRD42021257078

Review question

What are the factors, specifically barriers and facilitators, impacting implementation of self-management interventions in secondary care for people with severe mental illnesses?

Searches

The following databases will be searched from their inception to present date: MEDLINE (OVID SP), EMBASE (OVID SP), PsycINFO (OVID SP), CINAHL (EBSCOhost), and Web of Science Core Collection. The search will be limited to papers written in English. Additional searches will be performed through hand-searching and forward citation searching of the included papers to ensure all relevant papers are included. The search strategy was developed using terms relating to four domains (“self-management intervention”, “influencing factors”, “secondary care”, “severe mental illness”), combined using the Boolean operators (AND, OR). The terms were generated through a scoping review and by using the databases’ thesaurus.

Types of study to be included

Inclusion criteria:
Full-text journal articles which report on factors influencing implementation of a self-management intervention, for the population described above, and included primary data collection
Quantitative and qualitative studies

Condition or domain being studied

Factors impacting the implementation of self-management interventions for people with SMI

Participants/population [1 change]

Inclusion:
Adults (18 years and above) with SMI
Participants with a clinical diagnosis of schizophrenia (including schizophrenia-related disorders such as schizoaffective disorder, delusional disorder or psychosis), bipolar disorder, or major depression
Studies with mixed populations of people with SMI (at least 50% of participants must have a diagnosis of a SMI), using secondary mental health services
Exclusion:
Studies including only participants with unipolar depression, anxiety, personality disorders, or those with organic brain disorder or a primary diagnosis of substance abuse

Intervention(s), exposure(s)

The review will not investigate the effects of an intervention, but will explore the barriers and facilitators to the implementation of self-management interventions from the perspective of the patients and the clinicians who implement them. The setting must be in a secondary mental health care service.
Inclusion criteria for the intervention
The inclusion criteria have been created by Lean et al. (2019) and defines “self-management intervention” in the context of this review. The self-management intervention must include these three domains, which Mueser (2013) describes as “effective areas of self-management:”

1. Psychoeducation about mental illness and treatment
2. Recognition of early warning signs of relapse and development of a relapse prevention plan
3. Coping skills for dealing with persistent symptoms

Exclusion criteria for the intervention
Studies will be excluded if:

1. The intervention did not cover all three domains listed above.
2. The intervention involved basic psychoeducation that is not adapted to the individual.
3. The focus of the intervention was beyond improving an individual's self-management of their illness e.g. cognitive remediation, life skills or social skills.
4. The intervention was delivered to family members (either as sole recipients or in addition to the service users).
5. The self-management intervention was delivered as part of or alongside another intervention.

Comparator(s)/control

A comparator or control is not required.

Context

Secondary mental health care services.

Main outcome(s)

Any data on factors that impact the implementation of self-management interventions for people with severe mental illness in secondary mental health services.

Additional outcome(s)

None.

Data extraction (selection and coding)

Article screening and selection:
The titles and abstracts of the articles from the search will be screened by the main researcher to identify studies which appear to meet the inclusion criteria. A second researcher will screen 25% of titles and abstracts independently. The full text of these studies will be independently reviewed for eligibility. A second researcher will screen 25% of full texts of the chosen included studies. The reference lists of full-text articles selected will also be screened by the main researcher.
Data extraction:
The main researcher will extract data from the included studies. A second researcher will independently extract data from 20% of the included studies. Any disagreements will be discussed with a third researcher until a consensus is reached. The researchers will use a piloted document to extract the data which will include: study references (authors and date of publication), country, study design, duration of study, setting, participant details, limitations of the study, characteristics of the self-management intervention, implementation strategy, data collection and outcomes, barriers to the implementation and facilitators to the implementation.

Risk of bias (quality) assessment

A bias assessment will not be used, as this review is a qualitative synthesis. The main researcher will assess the quality of included studies using the Mixed Methods Appraisal Tool (MMAT) (Hong et al., 2018). This is to identify any weaknesses in the study design which may affect interpretation of the study findings, therefore, no effect size or score will be applied to the study.

Strategy for data synthesis

A narrative synthesis will be conducted to analyse the qualitative data, structured around the implementation of the interventions and the influencing factors. This will allow consideration of the contextual factors influencing implementation. A theoretical framework for locating factors influencing implementation of

interventions within conceptual domains, the Consolidated Framework for Implementation Research (Damschroder et al., 2009) will be used to guide the data analysis, as well as an established taxonomy of implementation outcomes (Proctor et al., 2011). This method will be piloted on five of the included studies and reviewed.

Analysis of subgroups or subsets
None planned.

Contact details for further information
Samihah Islam
samiha.islam.20@ucl.ac.uk

Organisational affiliation of the review
University College London
http://www.ucl.ac.uk

Review team members and their organisational affiliations
Miss Samihah Islam. University College London
Miss Rebecca Appleton. University College London
Professor Sonia Johnson. University College London
Dr Bryn Lloyd-Evans. University College London
Miss Chloe Hutchings-Hay. University College London
Miss Wing Tung Chung. University College London

Type and method of review
Narrative synthesis, Systematic review

Anticipated or actual start date
25 May 2021

Anticipated completion date
31 October 2021

Funding sources/sponsors
University College London

Conflicts of interest

Language
English

Country
England

Stage of review
Review Ongoing

Subject index terms status
Subject indexing assigned by CRD

Subject index terms
Humans; Mental Disorders; Quality of Life; Secondary Care; Self-Management

Date of registration in PROSPERO
27 May 2021

Date of first submission

25 May 2021

Stage of review at time of this submission

Stage	Started	Completed
Preliminary searches	Yes	Yes
Piloting of the study selection process	Yes	No
Formal screening of search results against eligibility criteria	No	No
Data extraction	No	No
Risk of bias (quality) assessment	No	No
Data analysis	No	No

Revision note

Erroneously omitted major depressive disorder from the inclusion of severe mental illness in the initial submission of the protocol

The record owner confirms that the information they have supplied for this submission is accurate and complete and they understand that deliberate provision of inaccurate information or omission of data may be construed as scientific misconduct.
The record owner confirms that they will update the status of the review when it is completed and will add publication details in due course.

Versions

27 May 2021
25 June 2021
